# Intestinal lipid droplets as novel mediators of host–pathogen interaction in *Drosophila*

**DOI:** 10.1242/bio.039040

**Published:** 2019-07-05

**Authors:** Sneh Harsh, Christa Heryanto, Ioannis Eleftherianos

**Affiliations:** Department of Biological Sciences, Institute for Biomedical Sciences, The George Washington University, Washington DC 20052, USA

**Keywords:** Lipid droplets, Infection, Midgut, *Drosophila*

## Abstract

Lipid droplets (LDs) are lipid-carrying multifunctional organelles, which might also interact with pathogens and influence the host immune response. However, the exact nature of these interactions remains currently unexplored. Here we show that systemic infection of *Drosophila* adult flies with non-pathogenic *Escherichia coli*, the extracellular bacterial pathogen *Photorhabdus luminescens* or the facultative intracellular pathogen *Photorhabdus asymbiotica* results in intestinal steatosis marked by lipid accumulation in the midgut. Accumulation of LDs in the midgut also correlates with increased whole-body lipid levels characterized by increased expression of genes regulating lipogenesis. The lipid-enriched midgut further displays reduced expression of the enteroendocrine-secreted hormone, Tachykinin. The observed lipid accumulation requires the Gram-negative cell wall pattern recognition molecule, PGRP-LC, but not PGRP-LE, for the humoral immune response. Altogether, our findings indicate that *Drosophila* LDs are inducible organelles, which can serve as markers for inflammation and, depending on the nature of the challenge, they can dictate the outcome of the infection.

## Abstract (precis)

**Summary:** Lipid droplets are inducible organelles, act as inflammatory markers and, depending on the nature of challenge, can dictate the outcome of the infection.

## INTRODUCTION

Lipid droplets (LDs) are specialized lipid-storing organelles which are found in almost all organisms ranging from bacteria to yeast and humans (Walther and Farese, 2009; Guo et al., 2009; Farese and Walther, 2009). LDs consist of a fatty acid monolayer and structural proteins surrounding a hydrophobic core of neutral lipids, mainly sterol and triglycerides (TGs) (Walther and Farese, 2009; Guo et al., 2009; Farese and Walther, 2009). In order to maintain energy homeostasis, a constant balance is maintained between the degradation and synthesis of lipids, where degradation is regulated by the Perilipin family of proteins (Plin1 and Plin2) while lipid biogenesis mainly involves a series of enzymatic reactions catalyzing the conversion of Fatty acyl CoA to complex TGs (Wilfling et al., 2014; Yen et al., 2008; Brasaemle, 2007).

LDs were originally shown to play a passive role in lipid homeostasis, however they are increasingly perceived as dynamic, multifunctional organelles. Their proteome contains key components that imply interactions with a variety of cell-specialized structures including mitochondria, endoplasmic reticulum and peroxisome (Beller et al., 2010b). Their presence in immune cells, in particular neutrophils and macrophages, indicates their role in regulating host–pathogen interactions and through modulating the host immune response (Melo and Weller, 2016; den Brok et al., 2018; Bozza et al., 2009; Weller et al., 1989). For instance, hepatitis C (HCV) and the dengue virus (DENV) infection in the hepatoma and kidney cell lines have been linked to enhanced lipogenesis and a sharp increase in LD numbers (Filipe and McLauchlan, 2015; Samsa et al., 2009). Although the mechanism of lipid accumulation is not known, it has been proposed that these viruses might reside in LDs to promote their own assembly and replication (Filipe and McLauchlan, 2015; Samsa et al., 2009). Infection of human monocyte cells and HeLa cells with the intracellular bacterial pathogens *Mycobacterium tuberculosis* and *Chlamydia trachomatis* also increases the number of LDs, which probably serve as energy and nutrient sources for the propagating bacteria (Nawabi et al., 2008; D'Avila et al., 2008; Mattos et al., 2011a,b; Daniel et al., 2011; Kumar et al., 2006). Furthermore, when peritoneal-and bone marrow-derived macrophages are infected with *Mycobacterium leprae*, *Mycobacterium* *bovis* or *Leishmania infantum chagasi*, LDs act as a source of prostaglandin and leukotriene eicosanoids, which are able to modulate inflammation and the immune response (Araujo-Santos et al., 2014; Mattos et al., 2011a, 2010; D'Avila et al., 2008).

In recent years, increasing pieces of evidence have demonstrated that *Drosophila* is a suitable model for dissecting lipid metabolism and energy homeostasis due to similarity with mammals in the type of organs and cells controlling metabolic functions and the conservation of signaling pathways involved in these processes (Kuhnlein, 2011, 2012). In *Drosophila*, *lsd-1/plin1* and *lsd-2/plin2* regulate lipolysis and both genes are well conserved in mammals. While *Lsd-1* and *Lsd-2* have contrasting functions and act in redundant fashion in *Drosophila*, in mammals, their function is still not clear yet. In *Drosophila*, storage lipids in the form of TGs and cholesterol ester are mainly accumulated in the adipose tissue (fat body) and partially in the intestine (gut) (Kuhnlein, 2012). Certain diseases including obesity, lipodystrophy, diabetes and neuronal disorders have been associated with impaired lipid homeostasis using the *Drosophila* model (Liu and Huang, 2013; Kuhnlein, 2011). In the context of immunity, there have been few, but compelling, cases implicating the role of LDs in host–pathogen interactions. Interestingly, the lipid-storing fat body and gut also form the primary immune organs in *Drosophila*, where fat body induces secretion of Toll and immune deficiency (Imd) signaling regulated antimicrobial peptides (AMPs), while the gut induces secretion of Imd regulated AMPs and reactive oxygen species (ROS) (Kuraishi et al., 2013; Charroux and Royet, 2010). *In vitro* and *in vivo* studies in *Drosophila* have revealed that histone bound to cytosolic lipid forms a cellular antibacterial defense system. In the presence of bacterial lipopolysaccharide, histones, which are normally sequestered into LDs, are released and eliminate the bacteria (Anand et al., 2012). In an attempt to establish a link between immunity and lipid metabolism, pathobiont-induced uracil production in *Drosophila* has been shown to play a critical role in distinguishing between harmful and commensal benign bacteria. In the presence of pathobionts, gut cells undergo uracil-induced metabolic switch, which in turn is required to sustain dual oxidase (DUOX) and ROS production in the enterocytes (Lee et al., 2018).

Despite previous reports in *Drosophila* proposing a link between immune function and LDs, a direct demonstration of infection-induced modulation in LD dynamics has not been found yet. For a more comprehensive understanding of the participation of LDs in host–pathogen interactions, we employed the potent pathogenic bacteria *Photorhabdus luminescens* and *Photorhabdus asymbiotica* (Enterobacteriaceae), which are able to interfere with humoral and cellular immune responses in *Drosophila* (Castillo et al., 2013; Aymeric et al., 2010), in order to induce systemic infection in adult flies and explore modulation in LD status. In terms of mode of infection and dissemination, *P. luminescens* is an extracellular insect pathogen while *P asymbiotica* is intracellular and acts as both opportunistic human pathogen as well as entomopathogen (Shokal and Eleftherianos, 2017; Duchaud et al., 2003; Waterfield et al., 2009).

Here we show that systemic infection with *Photorhabdus* bacteria induces intestinal steatosis marked by lipid accumulation and overall increase in systemic lipid levels. The intestinal steatosis is linked to increased lipogenesis, which in turn is regulated by the level of gut hormones. LD accumulation is mediated through Gram-negative cell wall recognition machinery, and accumulation of LDs can either provide resistance or be deleterious for the infected flies depending on the type of bacterial infection. Finally, infection-induced lipid accumulation can be mimicked upon genetic activation of Toll or Imd signaling pathways, suggesting that LD accumulation correlates with the activation of immune signaling pathways. These findings establish intestinal steatosis as one of the markers and regulators of the antibacterial immune response, which could open new avenues for clarifying the interrelationship between innate immunity and lipid metabolism.

## RESULTS

### Systemic bacterial infection in *Drosophila* adult flies results in intestinal steatosis

**Fig. 1.**
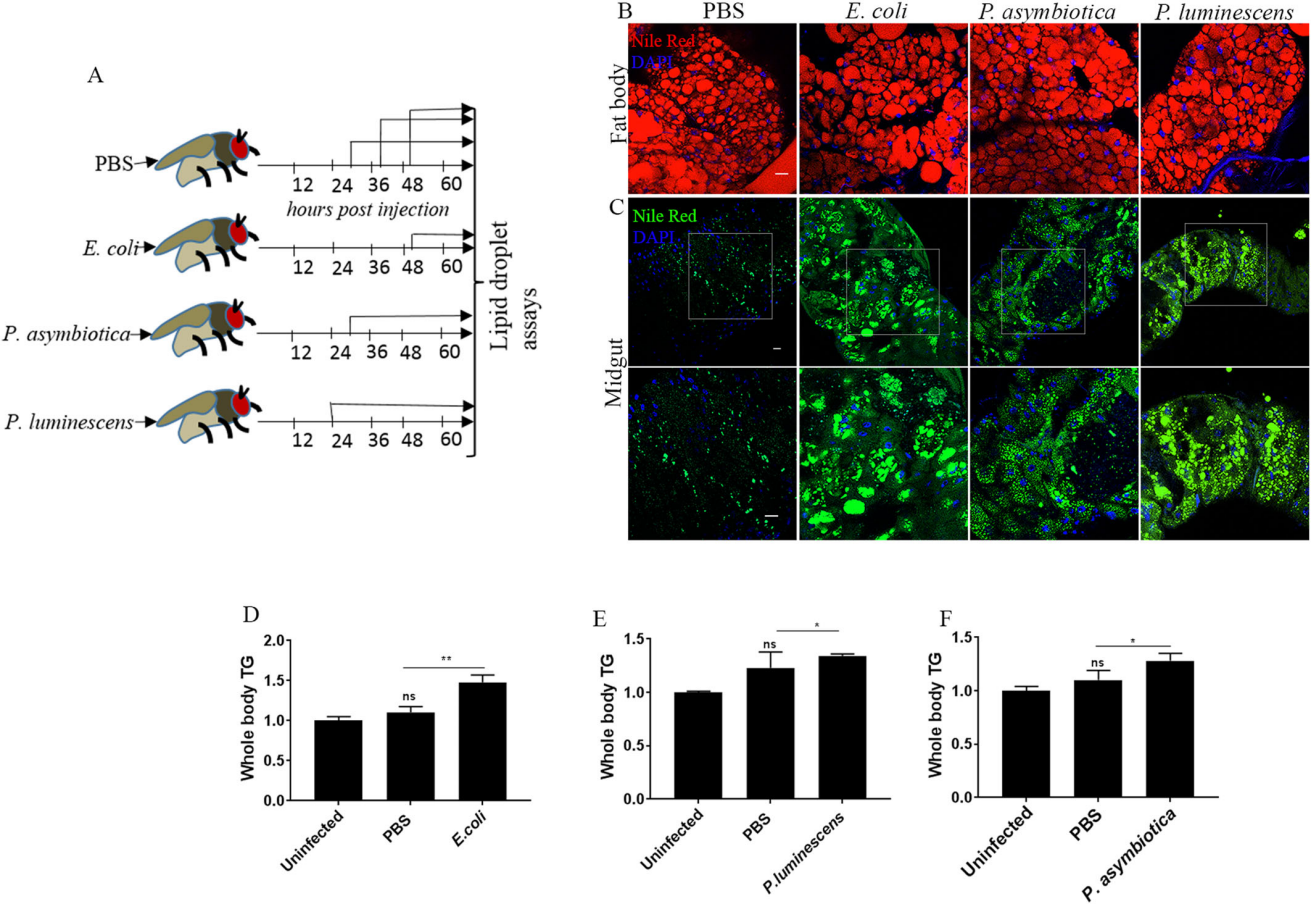
**Systemic bacterial infection results in midgut lipid accumulation and increased fly body lipid storage.** (A) Overview of the experimental workflow. *Drosophila melanogaster* background strain (strain *w^1118^*) flies were injected with PBS, *E. coli*, *P. asymbiotica* or *P. luminescens*, and fat body and midgut tissues were dissected to examine the status of LDs at 50, 30 and 24 hpi. (B) Representative images of fat body LDs from *w^1118^* flies injected with PBS or 100–300 CFU of *E. coli*, *P. asymbiotica* or *P. luminescens*. Injection with PBS served as negative control. There was no substantial difference in the size of LDs among the different types of bacterial infections compared to PBS-injected controls. Fat body LDs were visualized with the fluorescent dye Nile Red (red) and nuclei were tagged with DAPI (blue). (C) Representative images of midgut LDs from *w^1118^* flies injected with PBS or 100–300 CFU of *E. coli*, *P. asymbiotica* or *P. luminescens*. Bacterial infection resulted in dramatic accumulation of LDs in the midgut of the infected flies compared to PBS-injected controls. LDs were visualized with Nile Red (green) and nuclei with DAPI (blue). Lower panels show enlarged view of midgut LDs (outlined). (D–F) Systemic infection of background control flies with *E. coli*, *P. asymbiotica* or *P. luminescens* increased triglyceride levels in the fly body. Data represent the mean±s.d of three independent experiments. Asterisks indicate statistically significant differences compared to PBS-injected controls (Student's unpaired *t-*test*,* *P<0.05 and **P=0.005; ns, not significant). Scale bars: 100 μm.

The fat body and gut constitute the primary immune tissues of *Drosophila* (Buchon et al., 2014)*.* The fat body is responsible for secretion of the Toll and Imd signaling-mediated AMPs while the midgut mainly generates ROS and Imd-regulated AMPs (Broderick, 2016; Charroux and Royet, 2010; Liu et al., 2017). Interestingly, fat body and gut also form the primary metabolic organs in *Drosophila* (Arrese and Soulages, 2010; Liu and Jin, 2017; Song et al., 2014) and act as a reservoir for storing lipids. Given the close proximity of the lipids with these inflammatory organelles, our goal was to examine whether LDs could also act as mediators of immunity in *Drosophila*. We injected the thorax of background control *w^1118^* adult flies with 100–300 colony-forming units (CFU) of the well-characterized pathogens *P. asymbiotica* or *P. luminescens* (Hallem et al., 2007; Eleftherianos et al., 2010; Castillo et al., 2015; Shokal and Eleftherianos, 2017), and examined changes in size and number of LDs in the infected flies*.* Injection with *Escherichia coli* served as non-pathogenic control while PBS served as septic injury control. Infection of *Drosophila* with *P. asymbiotica* or *P. luminescens* resulted in increased mortality with 50% of the infected flies dying by 30 h (*P. asymbiotica*) and 24 h (*P. luminescens*) post infection (hpi), respectively (Fig. S1). Injection with non-pathogenic *E. coli* or sterile PBS did not affect fly survival (Fig. S1). Then, we estimated changes in size and number of LDs in the infected flies based on their survival rate. Thus, flies injected with *P. asymbiotica* or *P. luminescens* were processed for LD assessment at 30 or 24 hpi, respectively. For flies injected with the non-pathogenic *E. coli*, 50 hpi was chosen for estimating LD status while PBS-injected flies were checked at all time points (24, 30 and 50 hpi corresponding to the different types of bacterial infections) (Fig. 1A). We found that flies injected with *E. coli*, *P. asymbiotica* or *P.luminescens* showed no defect in fat body LDs as compared to flies injected with PBS (Fig. 1B and Fig. S2A,B). In contrast, systemic infection of adult flies with *P. asymbiotica* or *P. luminescens* resulted in dramatic accumulation of LDs in the midgut as compared to the PBS-injected flies, where LDs were distributed in a diffuse pattern (Fig. 1C). Interestingly, infection with non-pathogenic *E. coli* also resulted in midgut lipid accumulation compared to PBS-injected individuals (Fig. 1C). Intestines are instrumental in lipid mobilization. This is exemplified by the fact that under normal circumstances, intestinal triglyceride (TG) level, the major constituent of neutral lipid, accounts for only about 1% of the total body TG content. Abnormal retention of LDs in the midgut prompted us to estimate the status of TG storage in the infected flies. Indeed, we found that in agreement with the accumulation of LDs in the midgut, these flies also contained increased levels of TG (Fig. 1D–F). Thus, we conclude that systemic bacterial infection in *Drosophila* flies results in perturbed intestinal lipid metabolism marked by intestinal steatosis and overall increased systemic TG accumulation.

### Bacterial infection-induced lipid perturbation is associated with increased lipogenesis

**Fig. 2.**
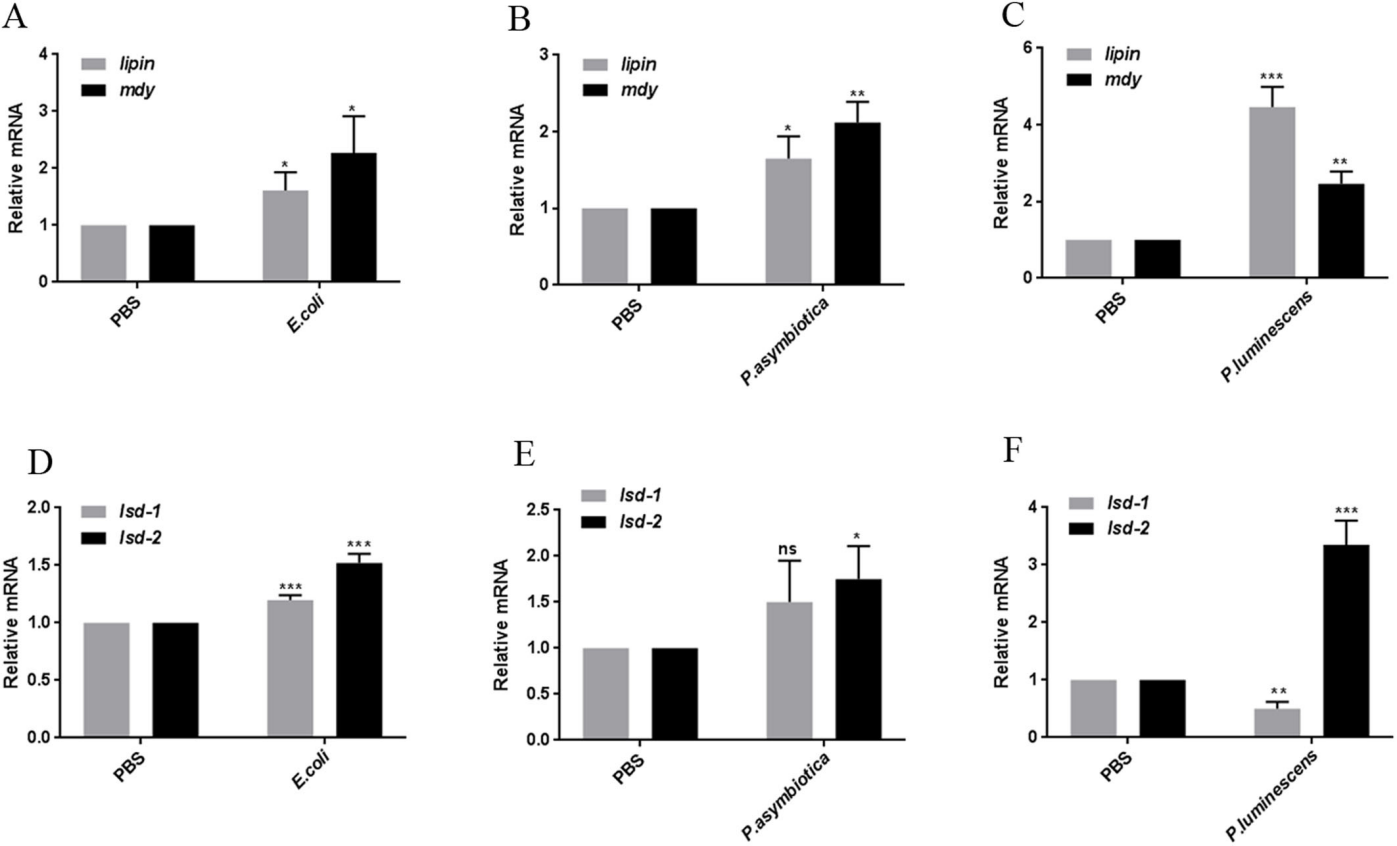
**Bacterial infection results in altered expression of genes regulating lipogenesis and lipolysis.** Background control flies (strain *w^1118^*) were injected with 100–300 CFU of *E. coli*, *P. asymbiotica* or *P. luminescens* and then frozen at 50, 30 and 24 hpi, respectively. The infected flies were processed for transcript level analysis of lipid-metabolism related genes. PBS-injected flies served as negative control. (A–C) mRNA level of genes involved in lipogenesis. (D–F) Expression of lipolysis related genes in the whole fly. (A–C) Flies infected with *E. coli*, *P. asymbiotica* or *P. luminescens* showed consistent upregulation of genes involved in lipogenesis, marked by the increased expression of *lipin* and *mdy.* (D–F) Unlike lipogenesis, the effect on lipolysis was distinct among the different types of bacterial infection. *lsd-1* and *lsd-2* were used as read-outs for lipolysis. While *lsd-1* was upregulated by *E. coli*, its level was reduced significantly upon infection with *P. luminescens*. *lsd-2* was significantly and consistently upregulated upon infection with *E. coli*, *P. asymbiotica* or *P. luminescens.* All mRNA levels were normalized against *RpL32* and three independent experiments were performed. Graphs depict the mean±s.d. Asterisks indicate statistically significant differences compared to PBS-injected controls (Student's unpaired *t-*test, *P<0.05, **P<0.005, ***P<0.001; ns, not significant).

We next examined the molecular basis of the bacterial infection-induced perturbation of lipid metabolism. The biosynthesis of TGs (the main constituent of LDs) is carried out through a series of enzymatic reactions converting fatty acyl-CoA to diacylglyceride (DG) and the final conversion of DG to TG (Coleman and Lee, 2004; Kuhnlein, 2012). The conversion to DG is facilitated by the phosphatidate phosphatase Lipin, while conversion of DG to TG is catalyzed by a diglyceride acyltransferase (DGAT), encoded by *Drosophila midway* (*mdy*) (Kuhnlein, 2012; Buszczak et al., 2002). Lipin and *mdy* act as major regulators of lipid storage in *Drosophila*; knockdown of *lipin* and *mdy* results in reduced lipid storage and increased lethality (Ugrankar et al., 2011; Beller et al., 2008). To test whether the enhanced lipid accumulation is linked to increased lipogenesis, we examined the mRNA expression of *lipin* and *mdy* in flies infected with bacteria. We found that flies challenged with *E. coli*, *P. asymbiotica* or *P. luminescens* had increased lipid biogenesis marked by significant enrichment of *lipin* and *mdy* as compared to PBS-injected flies (Fig. 2A–C). Further, infection with *E. coli* or *P. asymbiotica* induced a modest upregulation of *lipin* and *mdy* (Fig. 2A,B); however, infection with *P. luminescens* resulted in a robust upregulation of lipid biogenesis marked by 4.5- and 3-fold enrichment of *lipin* and *mdy*, respectively (Fig. 2C).

In contrast to lipogenesis, lipolysis entails breakdown of complex TG into DG and free fatty acids and thus makes TG metabolically accessible to tissues. In *Drosophila*, Perilipin-like domain-containing proteins (Lu et al., 2001) DmPLIN1 (Lsd-1) and DmPLIN2 (Lsd-2) modulate the rate of lipolysis (Gronke et al., 2003; Beller et al., 2010a; Teixeira et al., 2003). Lsd-1 is broadly expressed in fat body cell LDs and promotes lipolysis (Beller et al., 2010a; Bi et al., 2012). Lsd-2 functions opposite to Lsd-1 and protects TG stores in a dose-dependent manner (Bi et al., 2012). Unlike Lsd-1, Lsd-2 is strongly expressed in fly ovaries (Chintapalli et al., 2007), and microarray analysis indicates its expression in the adult fat body, gut and Malpighian tubules (Teixeira et al., 2003). We next examined the transcript levels of *lsd-1* and *lsd-2* in the infected flies. We found that *lsd-2* was significantly upregulated in flies infected with *E. coli*, *P. asymbiotica* or *P. luminescens* (Fig. 2D–F). In contrast, *lsd-1* showed an irregular expression pattern in bacterially infected flies. In particular, *lsd-1* was upregulated in flies infected with *E. coli* (Fig. 2D), while it was downregulated in flies infected with *P. luminescens* (Fig. 2F). There was no significant change in mRNA levels of *lsd-1* upon challenge with *P. asymbiotica* (Fig. 2E)*.* Together, these data show that bacterial infection-induced lipid accumulation is linked to the increased lipogenesis in *Drosophila* adult flies.

We next examined the functional significance of the lipid metabolism genes enriched upon infection. In particular, the role of Lipin in *Drosophila* adipose tissue development has been well characterized (Ugrankar et al., 2011). Being indispensable for the growth of the organism, mutation in *lipin* causes lethality, impaired eclosion and dystrophy of the fat body (Ugrankar et al., 2011). In order to overcome this caveat, we downregulated *lipin* (*UAS-Lipin RNAi*) using gut-specific *Esg-Gal4* (*EsgGa4>UAS-LipinRNAi*) and then examined the effect on overall TG level. We found that gut-specific downregulation of *lipin* did not affect the overall infection-induced TG level. In particular, we found no significant difference in the TG level of the control (*Esg-Gal4*) and *lipin* downregulated flies (*Esg>UAS-LipinRNAi*) when infected with *P. asymbiotica* or *P. luminescens* as compared to the PBS-injected counterparts (Fig. S3A). In the case of *E. coli* infection, however, we did notice that *lipin* knockdown prevented the increase in overall TG levels upon infection when compared to PBS injected controls (Fig. S3A). In retrospect, we checked the efficiency of RNAi and found that the gut-specific knockdown does not correlate with reduced mRNA level of *lipin* in this tissue (Fig. S3B). This was not surprising since the existing findings have implicated the role of Lipin mainly in the fat body and the gut-specific role is yet to be established (Ugrankar et al., 2011). Therefore, we then downregulated *lipin* in the fat body using *FB-Gal4* (*FB-Gal4>UAS-LipinRNAi*) and found that although there was no downregulation in the flies, the larval carcass showed significant reduction in *lipin* mRNA level (Fig. S3C,D). Thus, these findings suggest that although Lipin is instrumental in overall lipid metabolism, it is dispensable in regulating the TG level of infected flies. These findings also indicate the involvement of other gut-specific molecules in regulating the infection-induced lipid perturbation.

### Bacterial infection-induced lipid perturbation correlates with reduced expression of lipogenesis regulating *Tachykinin* and insulin signaling

As one of the critical organs regulating energy homeostasis, the *Drosophila* gut (similar to the mammalian intestine) is subject to direct neural control (Cognigni et al., 2011). In addition, the *Drosophila* gut may also be regulated by neuroendocrine organs secreting extrinsic hormonal signals or by its own peptides, produced by the enteroendocrine cells (EECs) (Lemaitre and Miguel-Aliaga, 2013; Cognigni et al., 2011; Reiher et al., 2011). Based on the similarity in developmental program between EECs and neurons, it is considered that midgut EECs may perform some of the neuronal functions, such as regulating the intestinal physiology, and transducing the intestinal/nutritional state to other parts of the insect (Takashima et al., 2011; Hartenstein et al., 2010; Lemaitre and Miguel-Aliaga, 2013). Recently, it was demonstrated that the EEC-secreted peptide hormone, Tachykinin (TK), negatively regulates intestinal lipogenesis, and consequently systemic lipid levels (Song et al., 2014).

**Fig. 3.**
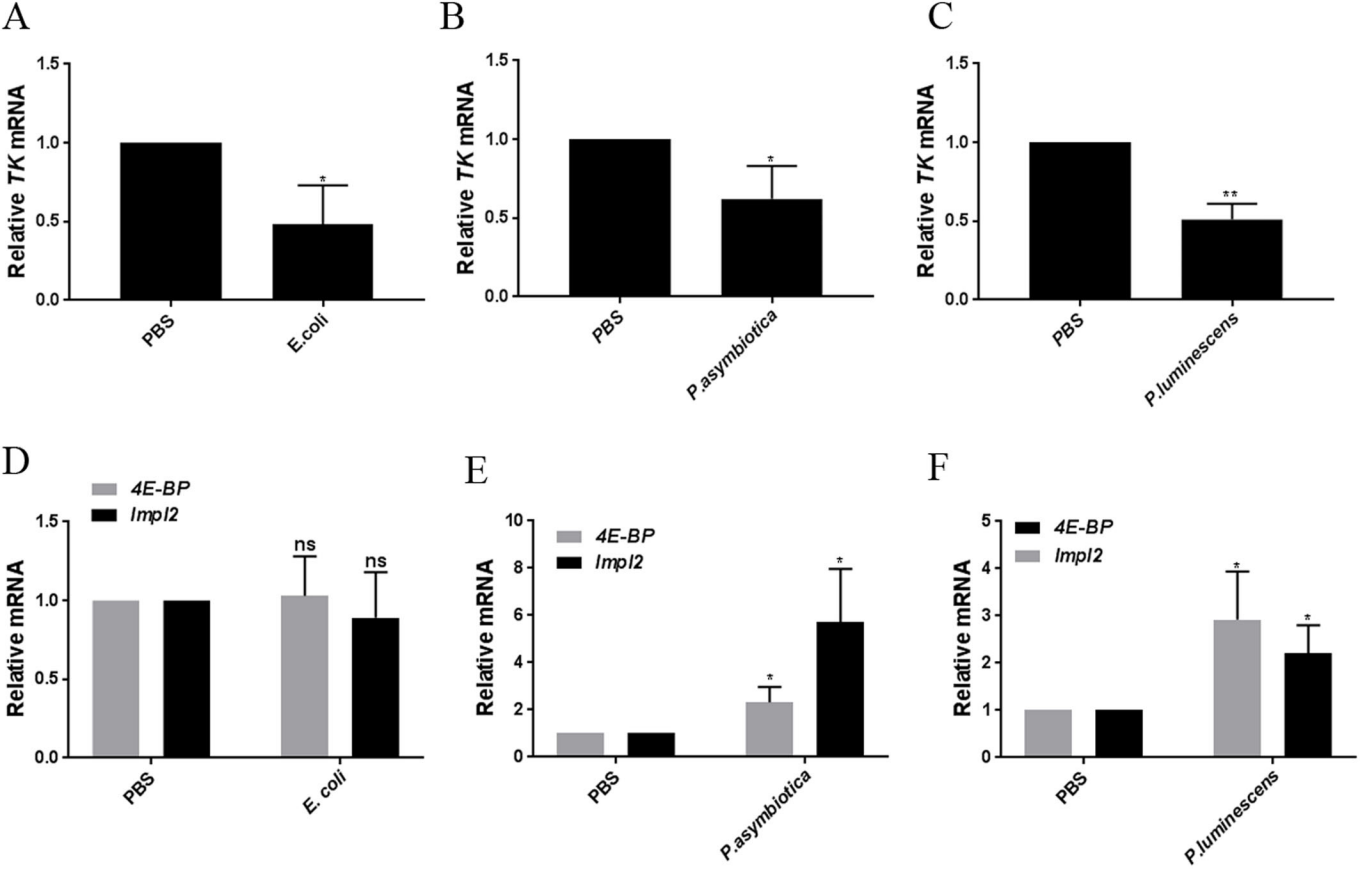
**Bacterial infection in *Drosophila* results in reduced expression of the gut-secreted hormone *Tachykinin* and insulin signaling.** Background control flies (strain *w^1118^*) were injected with 100–300 CFU of *E. coli*, *P. asymbiotica* or *P. luminescens* and dissected gut tissues were examined for mRNA expression of gut-secreting hormones and insulin signaling. PBS-injected flies served as negative control. (A–C) mRNA levels of gut-secreting hormone *TK*. (D–F) Expression of *4E-BP* and *Impl2*. (A–C) Flies infected with *E. coli*, *P. asymbiotica* or *P. luminescens* showed significantly decreased expression of *TK* as compared to the PBS-injected controls. *TK*-reduced levels of expression were consistent for all bacterial infections. (D–F) Gut tissues from flies infected with the pathogens *P. asymbiotica* or *P. luminescens* showed significant upregulation of *4E-BP* and *Impl2*, the negative regulators of insulin signaling. Infection with *E. coli* caused no altered expression of *4E-BP* and *Impl2*. Levels of mRNA were normalized against *RpL32* and three independent experiments were performed. Graphs depict the mean±s.d. Asterisks indicate statistically significant differences compared to PBS injected controls *(*Student's unpaired *t-*test*,* *P<0.05, **P=0.001; ns, not significant).

To characterize the contribution of TK in infection-induced lipid perturbation, we analyzed the mRNA expression levels of *TK* in the infected flies. We found that *TK* expression was significantly reduced in the gut of bacterially-challenged flies (Fig. 3A–C). The reduction was consistent for all bacterial infections. The significant reduction in *TK* expression further suggests the implication of gut hormones in modulating intestinal and systemic lipid levels upon bacterial infection.

The other prominent signaling pathway regulating metabolism is insulin signaling. Inactivated insulin signaling can lead to defect in lipid metabolism and enhanced level of fat storage (DiAngelo and Birnbaum, 2009). We found that the gut of flies infected with pathogenic *P. asymbiotica* or *P. luminescens* showed increased expression of *4E-BP* and *Impl2*, the negative regulators of insulin signaling (Honegger et al., 2008; Kwon et al., 2015), which indicated reduction in insulin activity (Fig. 3E,F). Upon infection with *E. coli*, no changes in expression of *4E-BP* and *Impl2* were observed (Fig. 3D).

These findings indicate that in addition to conveying the nutritional status, gut secreted neuropeptides may also be instrumental in controlling the pathological status of the fly through regulating lipid accumulation.

### DAP type peptidoglycan recognition protein PGRP-LC mediates bacterial infection-induced intestinal steatosis

**Fig. 4.**
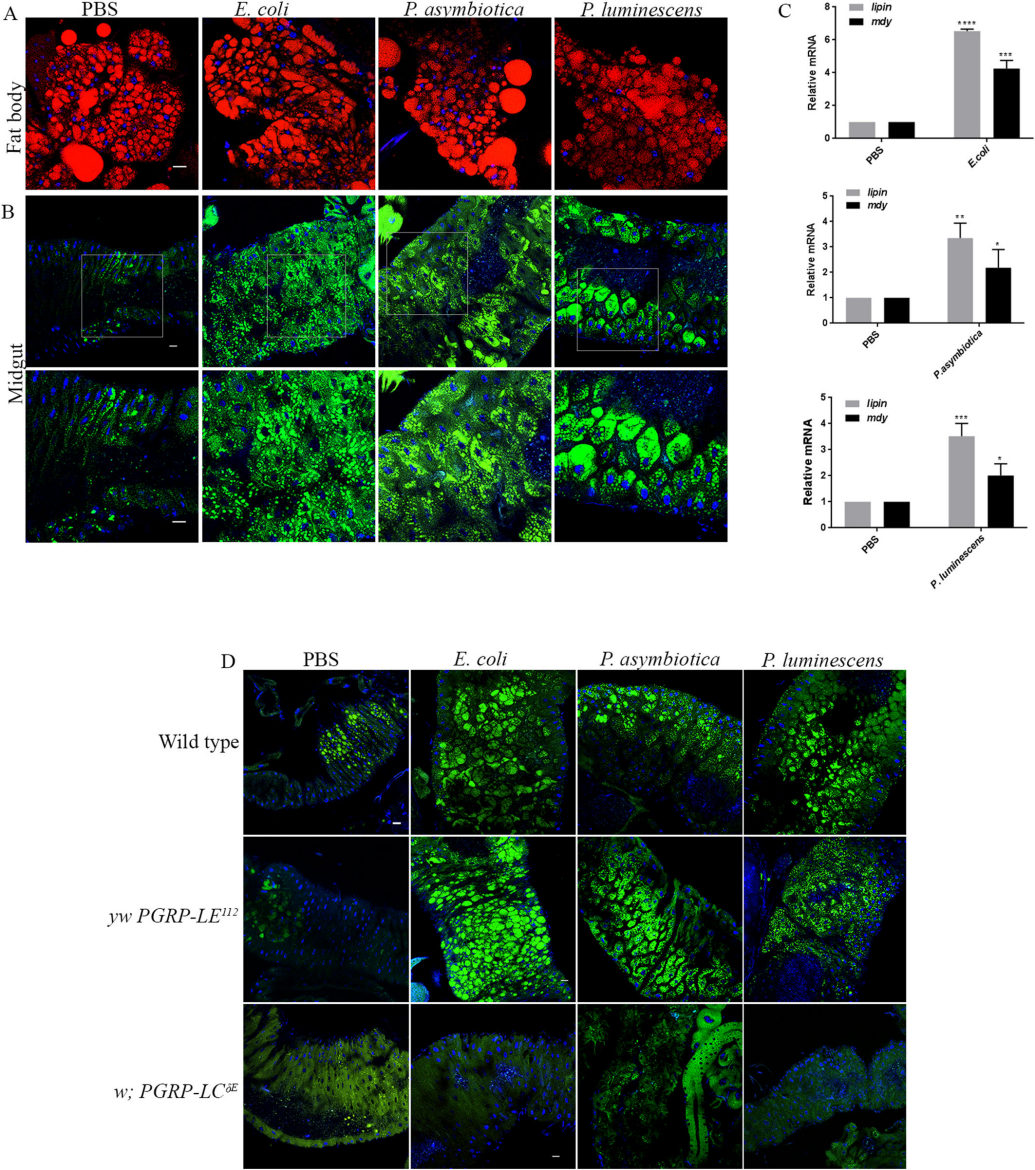
**Knockdown of the Gram-negative bacterial-recognition protein PGRP-LC ameliorates the bacterial infection-induced gut lipid accumulation.** (A) Representative images of fat body LDs from background control flies (strain *w^1118^*) injected with PBS or 100–300 CFU of heat-inactivated *E. coli*, *P. asymbiotica* or *P. luminescens*. Injection with PBS served as negative control. There was no noticeable difference in the size of LDs between treatments. Fat body LDs were visualized with the fluorescent dye Nile Red (red) and nuclei were stained with DAPI (blue). (B) Midgut tissues from flies injected with PBS or heat-inactivated *E. coli*, *P. asymbiotica* or *P. luminescens*. Midgut tissues from flies injected with heat-inactivated bacteria showed marked accumulation of LDs as compared to PBS-injected controls. Midgut LDs were visualized with Nile Red (green) and nuclei with DAPI (blue). Lower panels show the enlarged view of midgut LDs (outlined). (C) qRT-PCR revealed increased expression of genes regulating lipogenesis, *lipin* and *mdy* in flies injected with heat-inactivated *E. coli*, *P. asymbiotica* or *P. luminescens*. (D) Representative images of midgut LDs from background control flies and flies mutant for *PGRP-LE* (*yw PGRP-LE^112^*), *PGRP-LC* (*w; PGRP-LC^ΔE^*) upon injection with PBS, *E. coli*, *P. asymbiotica* or *P. luminescens.* Similar to the background controls (examined in both *yw* and *w^1118^* strains, but for simplicity representative images from *w^1118^* strain only are shown), *PGRP-LE* mutants showed dramatic accumulation of LDs in the midgut. In contrast, midgut tissues from PGRP-LC mutants did not show bacterial infection-induced lipid droplet accumulation following injection with *E. coli*, *P. asymbiotica* or *P. luminescens*. Midgut LDs were visualized with Nile Red (green) and nuclei were stained with DAPI (blue). Levels of mRNA were normalized against *RpL32* and three independent experiments were performed. Graphs show the mean±s.d. Asterisks indicate statistically significant differences compared to PBS-injected controls (Student's unpaired *t-*test*,* ****P<0.0001, ***P<0.05, **P=0.0023, *P<0.05). Scale bars: 100 μm.

Although not pathogenic to *Drosophila*, infection with *E. coli* resulted in increased accumulation of LDs in the midgut along with increased lipogenesis. The increase was as robust as in flies infected with the pathogens *P. asymbiotica* or *P. luminescens*. These observations prompted us to probe for the cellular mediators of lipid accumulation. Therefore, we examined lipid accumulation and the effect on lipid biosynthesis upon challenge with heat-inactivated bacteria. Similar to injection with live bacteria, we found that flies injected with heat-inactivated *E. coli*, *P. asymbiotica* or *P. luminescens* displayed enhanced lipid accumulation in the midgut (Fig. 4B). There was no noticeable defect in the fat body LDs (Fig. 4A). We further found that flies injected with heat-inactivated bacteria had increased lipid biosynthesis, marked by significant upregulation of *lipin* and *mdy* (Fig. 4C). Thus, these findings indicate that lipid accumulation is triggered through the recognition of certain pathogen-associated molecular patterns (PAMPs) of Gram-negative bacteria.

Pattern recognition in the *Drosophila* innate immune response relies largely on peptidoglycan (PGN) sensing by Peptidoglycan Recognition Proteins (PGRPs) (Werner et al., 2000; Stokes et al., 2015). While PGRP-SA and PGRP-SD recognize lysine-containing PGN produced by Gram-positive bacteria, PGRP-LC and PGRP-LE recognize Diaminopimelic acid (DAP)-type PGN, structures exclusive to Gram-negative bacteria (Stokes et al., 2015). Mutants for *PGRP-LC* and *PGRP-LE* are defective in eliciting an antimicrobial response and thus render these flies highly susceptible upon challenge with Gram-negative bacteria (Takehana et al., 2002; Gottar et al., 2002).

We next examined the contribution of PGRP-LC and PGRP-LE in mediating the infection-induced gut lipid accumulation. We injected flies mutant for *PGRP-LE* (*yw PGRP-LE^112^*) or *PGRP-LC* (*w; PGRP-LC*^ΔE^**)** with 100–300 CFU of *E. coli*, *P. asymbiotica* or *P. luminescens* and then estimated the effect on gut lipid accumulation. We noticed a dramatic increase in lipid accumulation in the midgut of *PGRP-LE* mutants and background control flies upon bacterial challenge (Fig. 4D). Interestingly, in contrast to *PGRP-LE*, we found no accumulation of LDs in the midgut of *PGRP-LC* mutants after infection with *E. coli*, *P. asymbiotica* or *P. luminescens* (Fig. 4D)*.*

These findings indicate that the Gram-negative sensing protein PGRP-LC mediates bacterial infection-induced intestinal steatosis.

### Intestinal steatosis confers a protective effect to flies infected with *P. asymbiotica* and sensitivity to flies infected with *P. luminescens*

**Fig. 5.**
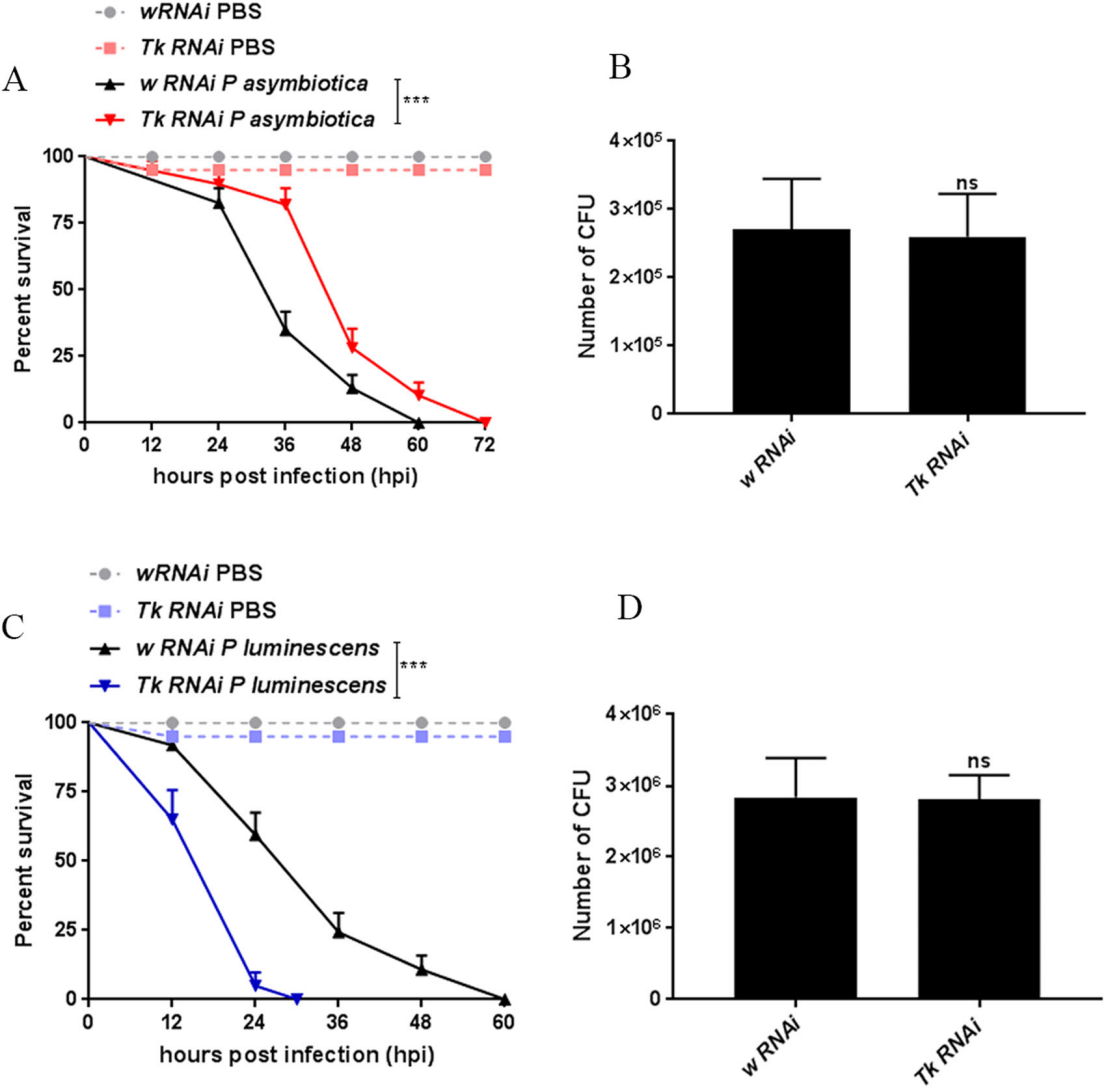
**Intestinal steatosis modulates the survival of bacterially infected flies without affecting bacterial load.** Survival and bacterial burden in flies knocked down for gut specific hormone TK driven under *TKg-Gal4* (*TKg>UAS-TK RNAi*) following intrathoracic injection with 100–300 CFU of *P. asymbiotica* or *P. luminescens*. Injection with PBS served as negative control. (A,B) *TK*-silenced flies (*TKg>UAS-TK RNAi*) survived longer as compared to control flies (*TKg>UAS-w RNAi*) when challenged with *P. asymbiotica*. While control flies reached 50% survival by 30 hpi, *TK*-silenced flies reached 50% survival by 40 hpi. (B) Quantification of bacterial burden in control flies (*TKg>UAS-w RNAi*) and flies with knocked down *TK* (*TKg>UAS-TK RNAi*) upon infection with *P. asymbiotica* (40 hpi). (C,D) *TK*-silenced flies (*TKg>UAS-TK RNAi*) were more sensitive to *P. luminescens* and succumbed at a faster rate as compared to the controls (*TKg>UAS-w RNAi*). Survival of *TK*-silenced flies and control flies dropped to 50% at 18 hpi and 24 hpi with *P. luminescens*, respectively. (D) Quantification of bacterial burden in control flies and flies with knocked-down *TK* upon systemic infection with *P. luminescens* (18 hpi). Log-rank (Mantel-Cox) was used to analyze the data (***P<0.0001). CFU were determined by qRT-PCR of *16SrRNA* against a standard bacterial curve and normalized against control flies (*TKg>UAS-w RNAi*). Three independent experiments were performed. Graphs show the mean±s.d. Statistical analysis was performed using Student's unpaired *t*-test (ns, not significant).

To test the functional significance of LDs in the context of bacterial infection, we chose genetic mutants bearing accumulated LDs in the midgut and increased systemic lipid levels. Downregulation of *TK* (*UAS-TK RNAi*) driven under the gut-specific driver *TKg*-*Gal4* (*TKg>UAS-TK RNAi*) has been shown to result in increased lipogenesis and LD accumulation in the gut (Song et al., 2014). We injected *TK*-silenced flies with *P. asymbiotica* or *P. luminescens* and then examined the effect on survival and bacterial load. Upon challenge with *P. asymbiotica*, *TK* knocked-down flies displayed prolonged survival as compared to control flies (Fig. 5A). Lipid accumulation slowed the mortality rate of *P. asymbiotica-*infected flies, which reached 50% survival by 40 hpi as compared to 30 hpi for the control flies. In contrast, infection of *TK*-silenced flies with *P. luminescens* displayed strong sensitivity, resulting in 50% survival by 18 hpi as compared to 24 hpi for the controls (Fig. 5C). *TK-*mediated lipid perturbation did not alter the survival rate of *E. coli*-infected flies (Fig. S4).

To investigate whether the modulation in survival is associated with changes in bacterial burden, we estimated bacterial load in the infected mutant strains. For this, we evaluated the number of CFU by qRT-PCR of *16SrRNA* against a standard bacterial curve and normalized against the background control strain. We found no changes in bacterial load in *TK*-silenced flies following infection with either *P. asymbiotica* or *P. luminescens* (Fig. 5B,D). Corresponding to the survival results, the bacterial load was estimated at 40 hpi for *P. asymbiotica* and 18 hpi for *P. luminescens.*

These results indicate that LDs in *Drosophila* can regulate the overall fitness against bacterial infection without affecting the bacterial burden.

### Immune signaling activation leads to defective lipid metabolism marked by enlarged fat body LDs and non-autonomous midgut lipid accumulation

The humoral arm of the *Drosophila* innate immune response mainly consists of the Toll and Imd signaling pathways, which regulate the induction of the downstream AMPs (Morin-Poulard et al., 2013; Buchon et al., 2014). Although for physiological infection-induced lipid phenotype we tested Gram-negative bacterial infections, we also explored the contribution of the different immune signaling pathways to lipid accumulation by testing the effect of genetic activation of Toll and Imd signaling on lipid accumulation. Interestingly, lipid modulation in the case of *M. tuberculosis* has been mainly attributed to Toll signaling activation (Barletta et al., 2016; Saitoh et al., 2011; Huang et al., 2014; Vallochi et al., 2018; Feingold et al., 2012)*.* Infection of *Drosophila* flies with *P. asymbiotica* or *P. luminescens* leads to upregulation of the Toll- and Imd-regulated *Drosocin* and *Cecropin* (Shokal and Eleftherianos, 2017). We examined whether the infection-induced modulation in LDs can be mimicked by genetic activation of immune signaling pathways. Toll and Imd signaling pathways were upregulated using the constitutively overexpressed constructs, *UAS-Toll^10b^* (Schneider et al., 1991) and *UAS-rel* (Vonkavaara et al., 2008) and the fat body-specific driver, *FB-Gal4* (*FB>UAS-Toll^10b^* and *FB>UAS-rel*) (Harrison et al., 1995). We noticed that activation of either immune signaling pathway resulted in enhanced lethality. The animals rarely eclosed and the majority died at the late larval stage (DiAngelo et al., 2009; Harrison et al., 1995; Qiu et al., 1998) (Table 1).

**Table 1.**
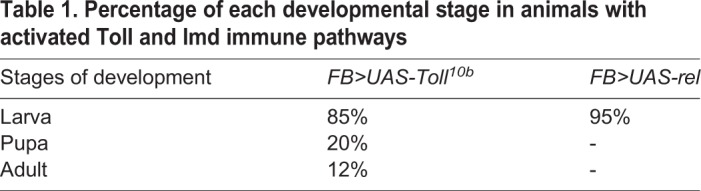
**Percentage of each developmental stage in animals with activated Toll and Imd immune pathways**

**Fig. 6.**
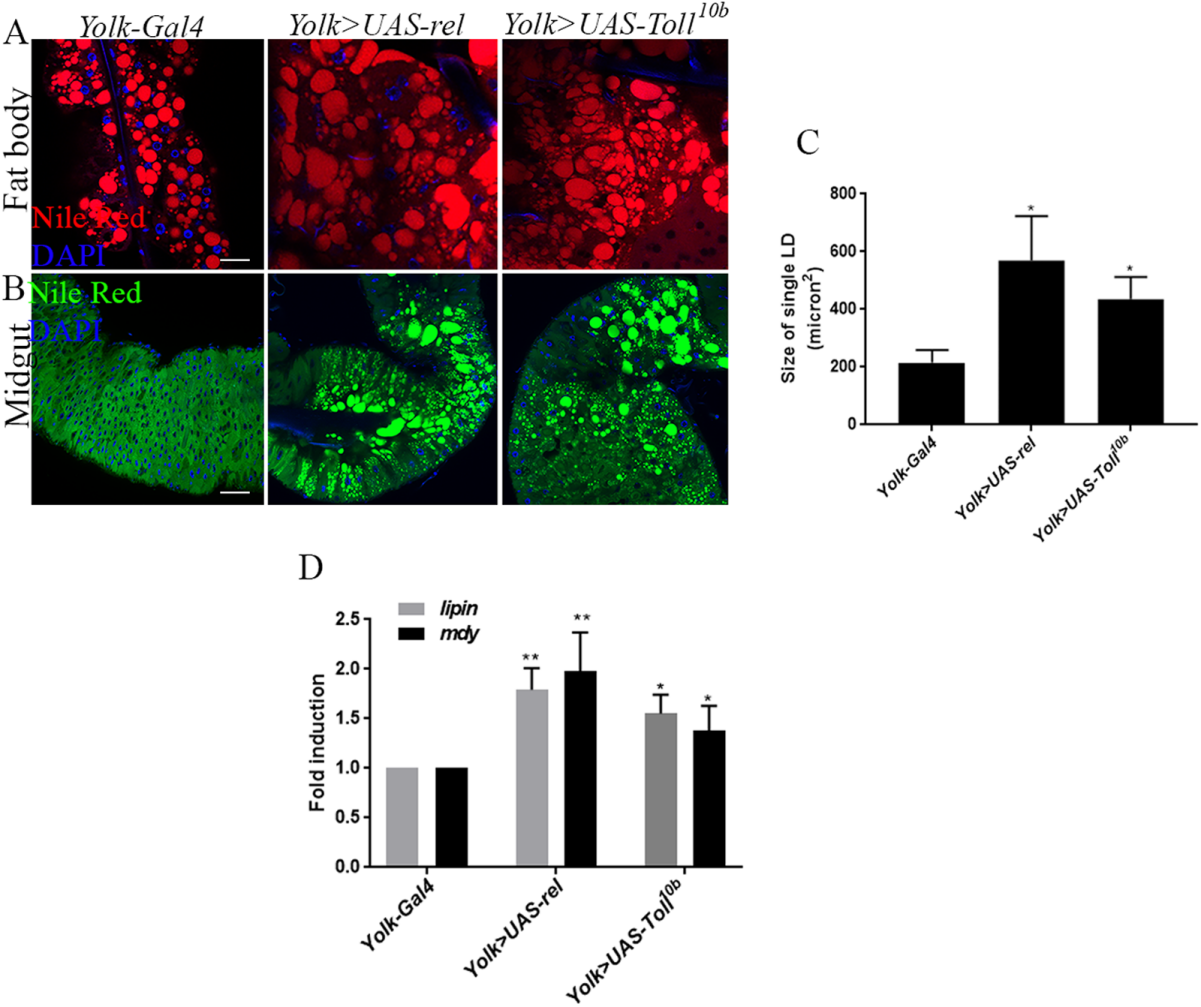
**Adult immune pathway activation results in localized enlarged fat body LDs and non-autonomous midgut lipid accumulation.** Toll and Imd signaling were constitutively activated in adult *D.* *melanogaster* and LD perturbation in the fat body and midgut were examined. Toll and Imd signaling were induced using the overexpression of activated Toll receptor *UAS-Toll^10b^* and overexpression of Relish (*UAS-rel*) under adult female fat body-specific driver *Yolk-Gal4* (*Yolk>UAS-Toll^10b^* and *Yolk>UAS-rel*), respectively. (A) Representative images of adult fat body LDs for the indicated immune signaling. LDs were marked with the fluorescent dye Nile Red (red), and nuclei with DAPI (blue). Adult flies with upregulated Toll or Imd signaling showed strikingly enlarged LDs in the fat body as compared to the control *Yolk-Gal4* strain. (B) Representative images of midgut LDs from flies carrying *Yolk-Gal4*, *Yolk-Gal4*-driven Toll or Imd overexpression (*FB>UAS-Toll^10b^*, *FB>UAS-rel*). Midgut tissues from adult flies overexpressing immune signaling pathways showed markedly increased accumulation of LDs compared to the control adult carrying *Yolk-Gal4* alone. LDs were visualized with Nile Red (green) and nuclei with DAPI (blue). (C) Quantification of fat body LD size in flies overexpressing immune signaling pathways. (D) qRT-PCR analysis showing increased transcript levels of lipogenesis-regulating genes *lipin* and *mdy* in the adult flies carrying overexpression of immune signaling pathways*.* Levels of mRNA were normalized against *RpL32* and three independent experiments were performed. Graphs depict the mean±s.d. Asterisks indicate statistically significant differences upon activation of immune signaling compared to *Yolk-Gal4* (Student's unpaired *t-*test*,* *P<0.05, **P<0.005). Scale bars: 100 μm.

In order to overcome this caveat, we used the *Yolk-Gal4* (Georgel et al., 2001), an adult female fat body-specific Gal4 driver, to induce the immune signaling pathways. Using *Yolk-Gal4*-driven *UAS-Toll^10b^* and *UAS-rel* constructs, we found that activation of immune signaling pathways in adult *Drosophila* was sufficient to trigger the lipid phenotype in a manner similar to the adult infection-induced lipid perturbation. However, as compared to the infected adult flies (where fat body failed to display lipid perturbation), we found that adult flies overexpressing Toll or Imd immune signaling unambiguously triggered enlargement of fat body LDs (Fig. 6A). Overexpression of Toll or Imd signaling resulted in 3–4 times increase in size of fat body LDs as compared to the control (Fig. 6C). In addition, adult flies with activated immune signaling also showed midgut lipid accumulation. As compared to the control, fat body-driven Toll and Imd overexpression triggered non-autonomous accumulation of the LDs in the midgut of the adult flies (Fig. 6B). Furthermore, consistent with the infection-induced lipid phenotype, flies carrying overexpression of Toll or Imd signaling showed significant increases in the expression of lipogenesis regulating genes *lipin* and *mdy* (Fig. 6D). These findings suggest that infection-induced lipid perturbation in *Drosophila* can be mimicked by constitutive activation of NF-κB immune signaling pathways.

## DISCUSSION

**Fig. 7.**
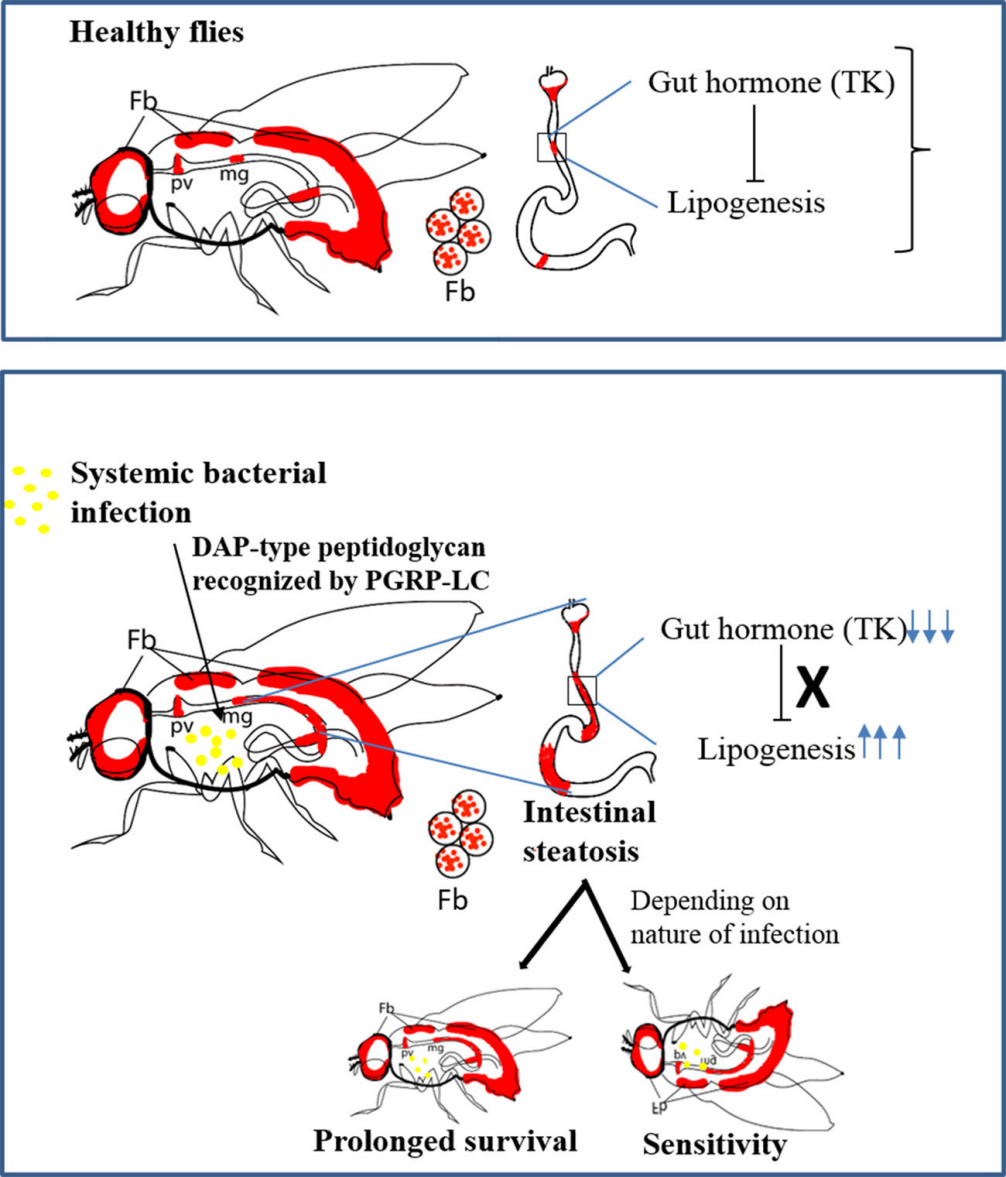
**PGRP-LC-mediated intestinal steatosis confers a protective or harmful effect in flies responding to bacterial infection.** (Upper panel) Scheme representing the host lipid dynamics in uninfected flies. LDs (red) are mainly localized in the fat body (Fb) and partly in proventriculus (pv) and midgut (mg) region of the gut. Gut hormone TK regulates lipid homeostasis in the gut as well as at the systemic level by suppressing lipogenesis. (Lower panel) Scheme representing the sequence of events triggered upon systemic bacterial infection. DAP-type peptidoglycan of Gram-negative bacteria is recognized by PGRP-LC, and this event is transduced in the form of intestinal steatosis marked by accumulation of LDs in the midgut of the infected flies without affecting fat body LDs. Intestinal steatosis is associated with reduced expression of TK, which in turn leads to increased rate of lipogenesis. The triggered intestinal steatosis can induce a protective or harmful response to the flies depending on the nature of bacterial infection.

LDs are increasingly recognized as a dynamic organelle and, other than lipid storage, have been assigned to interact with pathogens and thus affect host–pathogen interaction. However, owing to the complexity of the mammalian system, the role of LDs in host–pathogen interactions is still primitive. Using *Drosophila* as the model system, where the immune and metabolic signaling pathways are conserved with the mammalian system, we proposed to explore the host and infection-induced modulation in lipid dynamics in a more elaborate manner. We hypothesized that *Drosophila*, which is receptive to diverse challenges, could trigger the infection-induced lipid modulation as a sign of immunity. In order to have a comprehensive understanding of the role of LDs in host–pathogen interaction, we used three different bacterial infections and examined the response of the host in terms of the modulation in lipid dynamics. Here we show that systemic bacterial infection with *E. coli*, *P. asymbiotica* or *P. luminescens* in *Drosophila* flies results in intestinal steatosis marked by intestinal lipid accumulation without affecting the fat body LDs. Our results further show that the infection-induced lipid accumulation is associated with increased lipogenesis and enhanced systemic lipid levels. Expression analysis revealed the implication of gut hormone TK in inducing LD accumulation. In addition, we show that the DAP-type PGN recognition protein, PGRP-LC, is necessary for LD accumulation while PGRP-LE is indispensable. The infection-induced lipid accumulation is further mimicked by the overexpression of immune signaling pathways Toll and Imd in *Drosophila* adult flies. Finally, depending on the type of bacterial infection, LDs can be either beneficial or harmful to the infected host (Fig. 7).

A major progression in LD biology is the recognition of LDs as the inducible organelles, which can be elicited in response to inflammatory stimuli. Increased accumulation of LDs has been observed in a number of cell types and clinical cases including infected macrophages in atherosclerotic lesion (Schmitz and Grandl, 2008; Paul et al., 2008), granulomas during mycobacterial infection (Cardona et al., 2000), and leukocytes from patients with inflammatory arthritis (Bozza et al., 1996). LDs are thus increasingly perceived as structural markers for inflammation (Bozza et al., 2007). Apart from being induced in immune cells, LDs can also be induced in other organs, such as liver, which again forms a sign of inflammation. Accumulation in liver or hepatitis steatosis in particular, is prevalent and acts as a prognostic marker in HCV infection (Filipe and McLauchlan, 2015). In the *Drosophila* model, most LDs are localized in the adipose tissue equivalent, the fat body and a small proportion is found in the gut (Kuhnlein, 2011). Except for their central role in metabolism, fat body and gut also regulate immunity in *Drosophila*. In line with the correlation of LDs as markers for inflammation, our finding of infection-induced intestinal steatosis further validates that LDs in *Drosophila* are also inducible organelles and mediate a host-specific response upon infection. In addition, our finding that the infection-induced lipid perturbation could be mimicked upon genetic activation of immune signaling pathways further suggests that LDs can also act as inflammation markers. Indeed, more experiments will further elaborate on the specific role of LDs in the context of immune function. Absence of noticeable lipid perturbation in the fat body argues that in the case of *Drosophila*, it is the gut cells that respond to the presence of microbes and trigger the accumulation of LDs. Unlike mammals, *Drosophila* immune cells have not been reported to carry LDs and the absence of any evidence showing intimate association of hemocytes to gut further rules out the direct or indirect involvement of *Drosophila* immune cells in infection-induced gut lipid accumulation. Thus, our findings implicate intestinal steatosis as one of the reliable immune responses triggered upon systemic bacterial infection and genetic immune activation.

Brain, gut, endocrine gland and adipocytes form a complex signaling network that maintains energy homeostasis (Lemaitre and Miguel-Aliaga, 2013). Peptide hormones secreted from enteroendocrine cells in the gut, such as cholecystokinin (CCK), ghrelin and glucagon-like peptide 1 (glp-1) play a key role in this network. CCK, for instance, reduces food intake while ghrelin secretion reduces lipid mobilization in adipose tissues (Tschop et al., 2000; Sullivan et al., 2007). However, due to gene redundancy, loss-of-function studies in mouse have failed to show the cooperation between gut hormones and intestinal lipid metabolism. Similar to mammalian intestinal tract, the *Drosophila* adult gut secretes nine major gut prohormones which are processed into 24 mature peptides (Reiher et al., 2011). Interestingly, one of the most abundant peptides, TK, has been shown previously to regulate intestinal lipid homeostasis and hence systemic lipid levels (Song et al., 2014). Consistent with these findings, here we demonstrate that systemic bacterial infection-induced lipid accumulation is also associated with reduced expression of *TK.* Thus, our study reveals the physiological role of TK in the context of bacterial infection. To our knowledge, this is the first report implicating gut hormones in infection-induced lipid perturbation. Future investigations could focus on the molecular mechanisms promoting bacterial infection-induced downregulation of gut hormones, such as TK. It would also be interesting to explore the contribution of other gut hormones in the regulation of infection-induced lipid metabolism.

Elicitation of host immune responses initiate upon recognition of PAMPs by germ-line encoded receptors called pathogen recognition receptors (PRRs) (Stokes et al., 2015). In the case of tuberculosis, the cell-wall component of *Mycobacterium bovis*, trehalose-6,6′-dimycolate, caused an inflammatory response when coated in gel matrix and triggered lipid accumulation in macrophages or ‘foamy macrophages’ (Rhoades et al., 2005). Other mycobacterial cell wall components, such as oxygenated mycolic acids can also trigger LD accumulation in macrophages (Peyron et al., 2008). In case of DENV infection, it is the physical interaction of its replication machinery, the non-structural protein NS3 with fatty acid synthase which results in LD accumulation (Heaton et al., 2010). In correlation with these findings, here we show that the infection-induced intestinal steatosis is driven by the recognition of the DAP-type PGN, a characteristic component of Gram-negative bacterial cell wall (Stokes et al., 2015). DAP-type PGN, is recognized by two receptors, PGRP-LC and PGRP-LE. We found that while PGRP-LC is required for infection-induced lipid accumulation, PGRP-LE is dispensable in infection-induced intestinal steatosis. Importantly, the requirement of PGRP-LC for lipid accumulation was consistent for all bacterial infections. PGRP-LC and PGRP-LE have critical yet distinct functions in the *Drosophila* immune response to DAP type PGN. Although both receptors share the PGRP-domain, PGRP-LC is an extracellular receptor while PGRP-LE is a cytoplasmic intracellular receptor (Kaneko et al., 2006; Kurata, 2010). It remains to be shown whether this structural difference accounts for their distinct ability to induce LDs.

Although there are several instances of microbial infection-induced lipid accumulation, the exact function of LD accumulation in the context of infection has not been clarified. In case of HCV infection, LDs serve as sites for viral assembly, while in the case of *C. trachomatis* infection they act as a source of nutrients (Kumar et al., 2006; Filipe and McLauchlan, 2015). In contrast to these findings, LDs can form a source of pro-inflammatory eicosanoids or possess antimicrobial properties, such as viperin-mediated antiviral defense (Saka and Valdivia, 2012). In terms of infection with the intracellular pathogen *M. tuberculosis,* it was considered that the accumulated LDs are bacteria-derived, used as carbon source to facilitate bacterial propagation (Singh et al., 2012; Peyron et al., 2008). However, a recent study involving *in vitro* and *in vivo* infection demonstrated that *Mycobacterium*-induced LD formation is a programmed host response coordinated by cytokine IFN-γ, and LDs in turn act as source of host-protective eicosanoids (Knight et al., 2018). In correlation with these findings, our results demonstrate that LDs act as a double-edged sword that can be both harmful as well as beneficial to the infected host. Using two species from the potent pathogen *Photorhabdus*, we have shown that the outcome of accumulated LDs in *Drosophila* depends on the nature of infection. Thus, accumulated LDs provide prolonged survival to the flies upon infection with the facultative intracellular *P. asymbiotica*, while they confer sensitivity to flies upon infection with the extracellular *P. luminescens.* Future investigations will focus on the mechanistic basis that determines the function of accumulated LDs in *Drosophila* in the context of microbial infection.

In summary, we have provided an *in vivo* demonstration that bacterial infection and genetic activation of immune signaling pathways correlate with lipid perturbation marked by enhanced accumulation of LDs, indicating their implication in inflammation. At the upstream level, the function of PGRP-LC is indispensable for infection-induced lipid accumulation. Further, the transduction of PGRP-LC-mediated recognition to lipid accumulation is regulated via the gut hormone, TK. Survival results show that depending on the type of bacterial infection, LDs could be instrumental in determining the fate of the infected host. The current findings will contribute towards a better understanding of the participation of LDs in host–pathogen interactions.

## MATERIALS AND METHODS

### Fly stocks

The following fly lines were used: *w^1118^* (background control), *yw* (background control), *FB-Gal4* (Schmid et al., 2014), *tub-Gal4* (Bloomington Stock Center no. 5138), *yolk-Gal4* (Bloomington Stock Center no. 58814), *UAS-LipinRNAi (*VDRC transformant ID 36007*)*, *UAS-rel* (Bloomington Stock Center no.9459), *UAS-Toll^10b^* (Bloomington Stock Center no. 58987), *plin1^38^* (Bi et al., 2012), *UAS-plin1* (Bi et al., 2012), *UAS-TK RNAi* (Bloomington Stock Center no. 25800), *UAS-wRNAi* (Bloomington Stock Center no. 28980), *TKg-Gal4* (Song et al., 2014), *PGRP-LE^112^* (Bloomington Stock Center no. 33055), *PGRP-LC^ΔE^* (Bloomington Stock Center no. 55713). Genetic recombination was used to generate *UAS-plin1; tub-Gal4*.

### Bacterial strains

*E.* *coli* K12, *P.* *asymbiotica* subsp. *asymbiotica* (strain ATCC43949) and *P. luminescens* subsp. *laumondii* (strainTT01) were used for all fly infections. Bacterial cultures were prepared in sterile Luria-Bertani broth and maintained at 30°C for 18–22 h on a rotary shaker at 220 rpm. Bacterial cultures were pelleted down and then washed and resuspended in 1× sterile phosphate-buffered saline (PBS, Sigma-Aldrich). Bacterial concentrations were adjusted to an optical density (600 nm) of 0.015 for *E. coli*, 0.25 for *P. asymbiotica* and 0.1 for *P. luminescens* using a spectrophotometer (NanoDropTM 2000c, Thermo Fisher Scientific).

### Fly infection

Flies were reared on standard medium at 25°C. *w^1118^* or *yw* flies were used as background controls. Injections were performed by anesthetizing the flies with CO_2_. For each experiment, 5–6-day old adult flies were injected with bacterial suspensions using a nanoinjector (Nanoject III, Drummond Scientific). Heat-inactivated bacterial stocks were generated by exposing the bacterial inoculum to 56°C for 1 h in a water bath. Heat-inactivated or live bacterial solution (100–300 CFU) (18.4 nl) was injected into the thorax of flies and an injection of the same volume of PBS acted as negative control. Injected flies were then maintained at 25°C and processed for survival and other assays.

### Fly survival

For each fly strain, three groups of 20 female flies were injected with bacterial culture and one group was injected with PBS for control. Following injection, flies were maintained at a constant temperature of 25°C with a 12 h light/dark cycle, and survival was scored at 12-h intervals up to 72 h. Fly deaths occurring within 6 h of injection were attributed to injury and they were not included in the results. Log-rank (Mantel-Cox) was used to analyze the survival curves.

### qRT-PCR

Total RNA was extracted from 10 adult female flies at the indicated time points using Trizol according to manufacturer's protocol. Total RNA (500 ng–1 µg) was used to synthesize cDNA using the High Capacity cDNA reverse transcription kit (Applied Biosystems). qRT-PCR experiments were performed with technical triplicates and gene-specific primers in iQ SYBR Green Supermix (Bio-Rad) using a CFX96 Real-Time PCR detection system (Bio-Rad). Quantification was performed from three biological replicates for both test and control treatments. Primer sequences used in qRT-PCR assays were the following:

*RpL32* Forward: 5′-gatgaccatccgcccagca-3′, Reverse: 5′-cggaccgacagctgcttggc −3′; *Lsd-1* Forward: 5′-tgagccggcgacagcaacagt-3′, Reverse: 5′-cgtaggcggccgaaatggtg-3′ ; *Lsd-2* Forward: 5′-agtgtactagccgatacg-3′, Reverse: 5′-tctgactcccggatct-3′ ; *Lipin* Forward: 5′-gggcatgaatgaaatcga-3′, Reverse: 5′-tcaccaccttgtcgttgtg-3′ ; *Mdy* Forward: 5′-cgttctccaatatggacgtg-3′, Reverse: 5′-aaaagcagagccagcaaag-3′; *4E-BP* Forward: 5′-tcctggaggcaccaaacttatc-3′, Reverse: 5′-ggagccacggagattcttca-3′ ; *Impl2* Forward: 5′-aagagccgtggacctggta-3′, Reverse: 5′-ttggtgaacttgagccagtcg-3′ ; *P. luminescens 16S rRNA* Forward: 5′-acagagttggatcttgacgttaccc-3′, Reverse: 5′-aatcttgtttgctccccacgctt-3′ ; *P. asymbiotica 16S rRNA* Forward: 5′-gttacccgcagaagaagcac-3′, Reverse: 5′-ctacgcatttcaccgctaca-3′ ; *Tachykinin* Forward: 5′-tacaagcgtgcagctctctc-3′, Reverse: 5′-ctccagatcgctcttcttgc-3′.

### Bacterial load

Five adult flies of *w^1118^* strain were injected with *E. coli*, *P. asymbiotica* or *P. luminescens* and then frozen at 50, 30 and 24 h post injection. Total RNA was extracted from 10 adult female flies using Trizol according to manufacturer's protocol. Bacterial copy numbers were estimated by using primers against *16SrRNA*. Absolute copy numbers of bacteria were extrapolated by a standard curve constructed of six-point dilution series of bacterial DNA. All samples were run in technical triplicates and the experiments were repeated three times.

### Nile Red staining of neutral lipids and imaging

Fat body and gut tissues were dissected, fixed in 4% Para-formaldehyde in PBS for 30 min at room temperature. Fixed tissues were then rinsed twice in PBS, incubated for 30 min in 1:1000 dilution of 0.05% Nile Red prepared in 1 mg/ml of Methanol, and finally mounted in Antifade mountant with DAPI. To quantify LD size, the area of the 10 largest LDs per fat body cell was measured using ImageJ. This was repeated in at least three independent samples for each fly strain. Images were acquired with Zeiss LSM 510 confocal microscope and processed using Adobe Photoshop CS6.

### Triglyceride assay

Adult flies (*n*=15) were injected with *E. coli*, *P. asymbiotica*, *P. luminescens* or PBS and collected at 50, 30 and 24 h post injection. Groups of flies were washed and samples were prepared for colorimetric assays of triglyceride as previously described (Tennessen et al., 2014; McCormack et al., 2016). All samples and standards were run in triplicates and at least three independent experiments were performed. Triglyceride levels were normalized to total protein content present in the sample.

### Statistical analysis

An unpaired two-tailed Student's *t*-test was used for statistical analysis of data with GraphPad Prism (GraphPad Software). *P*<0.05 was considered statistically significant.

## Supplementary Material

Supplementary information
