# Supplementary material for: Intestinal lipid droplets as novel mediators of host–pathogen interaction in Drosophila
Source: Biol Open. 2019 Jul 5;8(7):bio039040. doi: 10.1242/bio.039040 (PMC6679391; doi:10.1242/bio.039040)
Supplement: Supplementary information [file biolopen-8-039040-s1.pdf]

Intestinal lipid droplets as novel mediators of host-pathogen interaction in *Drosophila*

Sneh Harsh, Christa Heryanto and Ioannis Eleftherianos[1]*

[1]Department of Biological Sciences, Institute for Biomedical Sciences, The George Washington University, Washington DC, United States of America

*** Corresponding author:** ioannise@gwu.edu (IE)

Biology Open • Supplementary information

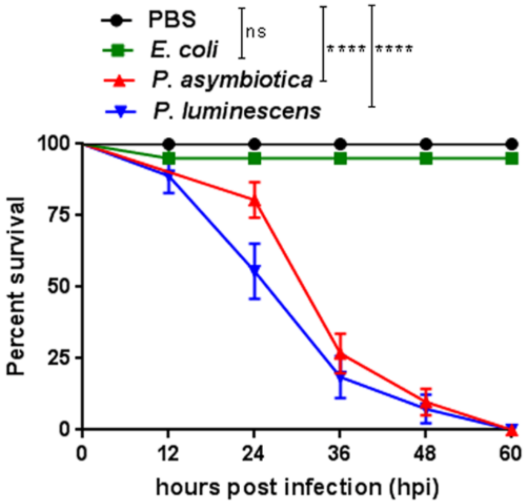

Fig. S1. Survival rate of w^{1118} flies injected with pathogenic or non-pathogenic bacteria. Flies of the w^{1118} strain were injected with 100-300 CFU of *E. coli*, *P. asymbiotica* or *P. luminescens* and survival was monitored every 12 hours and up to 72 hpi. Injection with PBS served as negative control. PBS and *E. coli* injections caused no effect on fly survival. Black and green lines depicting the survival of PBS and *E. coli* injected flies are superimposed, but for clarity, they are shown in parallel. w^{1118} flies were sensitive to *P. asymbiotica* (red line) and *P. luminescens* (blue line) infection. Infection with *P. asymbiotica* resulted in 50% lethality by 30 hpi as compared to infection with *P. luminescens*, where 50% of the flies died by 24 hpi. Log-rank (Mantel-Cox) was used to analyze the data (****p<0.0001; ns, not significant).

Biology Open • Supplementary information

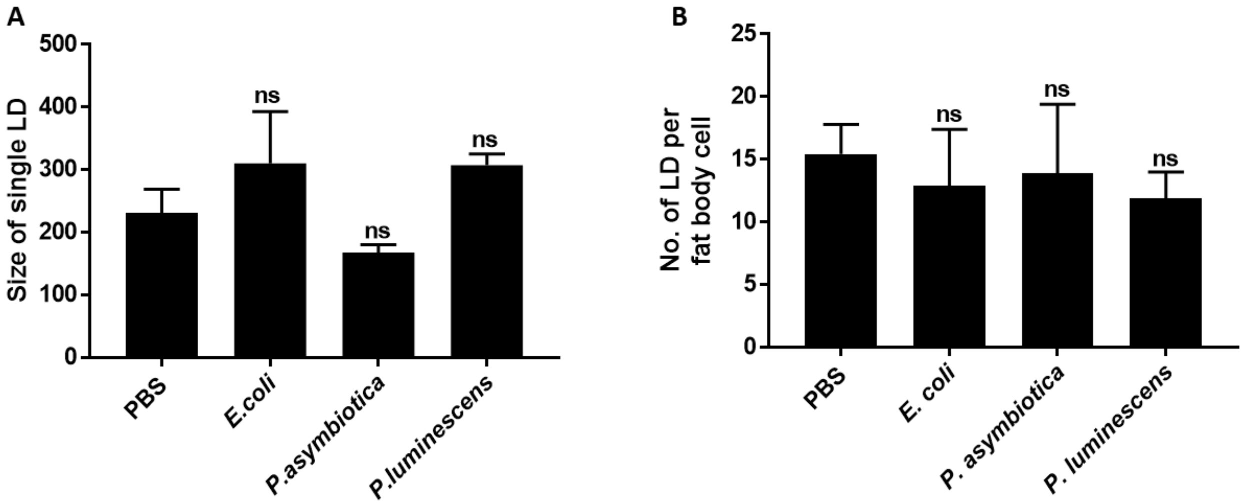

Fig. S2. Quantification of fat body lipid droplet size and number in bacterially infected flies. Quantification of fat body lipid droplet (A) size and (B) number in w^{1118} flies injected with *E. coli, P. asymbiotica* or *P. luminescens*. Injection with PBS served as negative control. Data represent the mean value ± s.d of three independent experiments. Student's unpaired t-test was performed to indicate statistically significant differences (ns, not significant).

Biology Open • Supplementary information

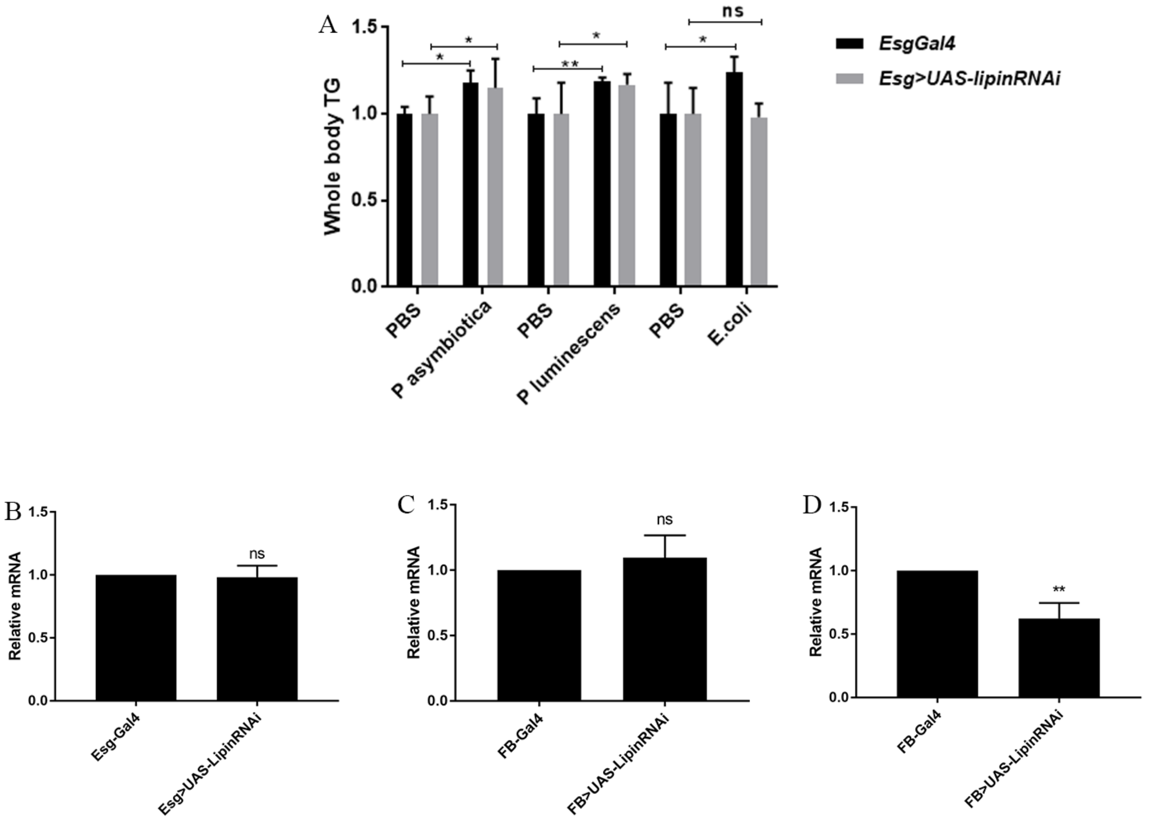

Fig. S3. Triglyceride (TG) level in bacterial infected control flies and flies carrying gut-specific *lipin* knockdown. (A) Whole body TG level in control flies (*Esg-Gal4*) and flies carrying gut-specific *lipin* knockdown (*Esg>UAS-lipinRNAi*) upon injection with PBS, *P. asymbiotica*, *E. coli* or *P. luminescens*. The TG level in flies carrying *lipin RNAi* (*Esg>UAS-lipinRNAi*) were comparable to those in control flies (*Esg-Gal4*) when infected with either *P. asymbiotica* or *P. luminescens*. The values for TG were normalized against the PBS injected flies of the respective genotypes. In case of infection with *E. coli*, there was no increase in TG level when *lipin* was downregulated. (B-D) qRT-PCR based mRNA level of *lipin* when *UAS-Lipin RNAi* was driven under *Esg* and *FB-Gal4*. Level of *lipin* mRNA was detected in the gut (B), whole flies (C) and larvae (D) of the respective genotypes. All mRNA levels were normalized against *RpL32* and three independent experiments were performed. Data represent the mean value ± s.d. Asterisks indicate statistically significant differences compared to PBS injected controls (Student's unpaired t-test, *p= 0.0180, 0.032, 0.0151, 0.0135 and **p=0.0018, 0.0049; ns, not significant).

Biology Open • Supplementary information

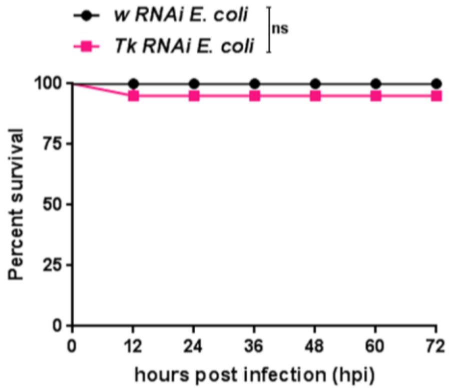

Fig. S4. Survival rate of control and *TK* silenced flies injected with non-pathogenic bacteria. Survival in flies knocked down for gut specific hormone Tachykinin driven under *TKg-Gal4 (TKg>UAS-TK RNAi)* following intrathoracic injection with 100-300 CFU of non-pathogenic *E. coli* as compared to the control flies *(TKg>UAS-w RNAi)*. Survival was monitored every 12 hours and up to 72 hpi. There was no difference in the survival rate between the two fly strains upon injection with *E. coli*. Black and magenta lines depicting the survival of *E. coli* injected control and *TK* silenced flies are superimposed, but for clarity, they are shown in parallel. Log-rank (Mantel-Cox) was used to analyze the data (ns, not significant).

Biology Open • Supplementary information
